# Comparison of tensile bond strength of ball attachments made of different materials to root canal dentin after chewing simulation

**DOI:** 10.1186/s12903-022-02282-3

**Published:** 2022-06-24

**Authors:** Ahmed Mostafa Abdelfattah Mohamed, Mahmoud El-Moutassem Bellah El Homossany, Sarah Mohamed Abdelmoniem, Tariq Yehia Abdelrahman

**Affiliations:** 1 Oral and Maxilofacial Prosthodontics Department, Faculty of Dentistry, AinShams University, Organization of African Unity Street, Cairo, 11561 Egypt; 2 Operative Dentistry Department, Faculty of Dentistry, AinShams University, Cairo, Egypt; 3 Endodontics Department, Faculty of Dentistry, AinShams University, Cairo, Egypt

**Keywords:** Overdenture, Ball attachment, Polyetheretherketone, Polyetherketoneketone, Tensile bond strength, Chewing simulation

## Abstract

**Background:**

Debonding of ball attachments is one of the complications that annoy teeth supported overdenture wearers. The polyetheretherketone (PEEK) and polyetherketoneketone (PEKK) polymers are widely applied in the dental field. The purpose of the current study was to compare the tensile bond strength of ball attachments made of such materials and the commonly used titanium ones after 5 years of overdenture insertion and removal (5000 cycles) in addition to chewing simulation (1,200,000 cycle).

**Methods:**

Extracted mandibular canines (N = 60) were randomly allocated into three groups and received ball attachments; titanium (group TI; N = 20), PEEK (group PE; N = 20), PEKK (group PK; N = 20). In each group, the samples were divided into two subgroups whereas tensile bond strength was measured pre aging (T0; n = 10) and post aging (T1; n = 10). Tensile bond strength was measured by the Pull out test using the Universal testing machine. Failure mode analysis was determined by examination of the samples’ surfaces under 65X stereomicroscope. The resulting data followed normal distribution and the significance level was set at (α = 0.05).

**Results:**

One Way Anova showed statistically significant difference between the three groups (P < .00001). PostHoc Tukey test showed statistically significant difference between the groups TI and PE, TI and PK and no statistically significant difference between the groups PE and PK. Paired t test showed statistically significant difference in the tensile bond strength pre and post aging in each group.

**Conclusions:**

PEEK and PEKK ball attachments could be concluded to have a higher tensile bond strength compared to the titanium ones when bonded to root dentin. Tensile bond strength of such attachments may decrease with aging as well. Clinically, the higher tensile bond strength may have a lesser rate of debonding and thus reduced patient apprehension.

## Background

Although the rate of complete edentulism may be declining, yet the remaining teeth in the elderly may act as poor abutments for conventional clasp retained removable partial denture [1]. Such remaining teeth may have compromised periodontal support resulting from aging and senility in addition to the effect of possible systemic condition. Shortening the clinical crown of the remaining teeth to serve as overdenture abutments would help to improve the crown-root ratio and reduce their mobility [2]. Thanks to the periodontal ligament receptors, the retained proprioception function improved the chewing efficiency and reduced the electromyographic activity in the masseter muscle [3–5]. The loss of sensory feedback in complete denture wearers was also accompanied by decrease in vertical dimension compared to the overdenture wearers. Such a decreased vertical dimension led to frontal and upward rotation of the mandible resulting in mandibular prognathism [5]. Moreover, the rate of bone resorption in overdenture wearers is less than in complete denture wearers [6]. On the other hand, overdenture wearers may experience some complications. Abutment teeth loss, denture stomatitis, recurrent caries and periapical lesions are examples of biological complications that may develop. However, mechanical complications may occur as debonding of the attachment, its fracture, wear of the matrix and denture base fracture [7–9].

Debonding of attachments was reported to occur as a result of removal and insertion of overdentures on daily basis to avoid tissue inflammation and denture stomatitis [8, 10]. Recementation of debonded attachments may not occur as in their previous positions resulting in misfit, attachment overloading and consequent fracture. Thus, it was recommended that the female housings should be removed from the overdenture and repicked up [8]. Several factors may affect the retention of the attachment in the root canal as the length of the attachment post, type of cement used [11, 12], surface treatment before cementation [13, 14] and material of the substrate [15, 16].

Polyetheretherketone (PEEK) and Polyetherketoneketone (PEKK) are members of high performance polyaryletherketones (PAEK). They have excellent biological, mechanical and physical properties for biomedical application. Shock absorption, excellent biocompatibility and modulus of elasticity similar to bone and dentin are further advantages. However, PEKK has better mechanical properties and higher fatigue limit compared to PEEK due to the extra ketone group [11, 12]. Such materials could be used in the dental field for fabrication of dental crowns, removable partial dentures, dental implants, obturators, root canal posts and attachments [16–18]. The modulus of elasticity of PAEK materials being close to root dentin in addition to their shock absorption make them suitable materials to be used as root canal posts. PAEK root canal posts allowed better stress distribution and less incidence of root fractures compared to titanium posts as reported in the literature [19].

Different techniques for cementation of PEEK and PEKK substrates are mentioned in the literature. Air abrasion with alumina particles, sulphuric acid etching, piranha acid etching and silanization were evaluated for bond strength as surface pretreatments before cementation. Different adhesive systems were tested as well [11–16, 20–23]. Schmidlin et al., Uhrenbacher et al. and Kern et al. reported lower tensile bond strength values for the unconditioned PEEK substrates and those with Piranha acid etching compared to those pretreated with sulphuric acid or air borne abrasion [13, 20, 22]. Although Furhmanna et al. reported improved bond strength values for PEEK substrates with silica coating, Hallmann et al. stated that there was no synergistic effect for silica coating on the tensile bond strength when coupled with air abrasion [14, 15]. Furthermore, Hallmann et al. recommended air abrasion of PEEK substrates before any chemical treatment. They stated that chemical etching neither makes enough roughness nor it increases the surface contact area as air abrasion does [14]. Moreover, Uhrenbacher et al. examined the effect of different adhesive systems as Visiolink, Signum bond and Ambarino. Such systems had comparable results in the presence of sulphuric acid etching or air borne abrasion pretreatments. Uhrenbacher et al. also pointed out the importance of PEEK pretreatment regardless of the adhesive system used [20]. Similarly, Song et al. reported higher tensile bond strength for sandblasted PEKK posts compared to the non pretreated ones. Moreover, silica coating did not have an additional effect on tensile bond strength coupled with sandblasting pretreatment for PEKK substrates [24]. However, these studies evaluated the bond strength of dental crowns and posts made of PEEK and PEKK but did not evaluate that of ball attachments used in tooth supported overdentures especially that debonding of commercially available ones are mentioned in the literature. Moreover, they adopted thermocycling only for aging and did not investigate the effect of mechanical aging in the form of dynamic simulated chewing in addition to the insertion and removal of overdentures by patients on the tensile bond strength of PEEK and PEKK substrates. So, the aim of this study was to evaluate the tensile bond strength of stud attachments made of Titanium, PEEK and PEKK when bonded to root dentin under simulated mechanical aging. The assumed null hypotheses was that the three materials had no difference in their tensile bond strength when used as stud attachments bonded to root dentin.

## Methods

### Sample size determination and grouping

Sixty extracted human mandibular canine teeth with no signs of internal or external resorption were used. Mature apex and absence of carious lesions or root canal fillings were mandatory as well. Samples were randomized and allocated into three equal groups according to the material of the ball attachment used; eighteen teeth (N = 20) in each group. In group TI, Titanium ball attachments were used and served as a control group. However, in group PE, they were made of PEEK and in group PK they were made of PEKK. In each group, the teeth were divided equally into two subgroups (n = 10); subgroup T0 and subgroup T1. The tensile bond strength in subgroup T0 was measured before aging while in subgroup T1, it was measured after aging.

The sample size was determined in the light of the results published by Fuhrmann et al. and Benli et al. [15, 16]. The sample size was calculated based on 95% confidence interval and power 90% with α error 5% (G.power 3.19.2) Ethical approval was granted by the ethical committee in the author’s university (RecER022221). The extracted teeth were taken from the archives of the Oral and Maxillofacial surgery department in the author’s university.

### Abutment teeth preparation

A single operator prepared the teeth and made the root canal treatment. The length of the roots was standardized to 15 mm using a diamond disc mounted in a low-speed hand piece under water coolant. The patency of the canal was validated using a Size # 10 K file (Mani, Utsunomiya, Japan). The working length was shorter 1 mm from the length obtained when the file tip just appeared at the apical foramen. Root canals were instrumented using ProTaper Gold rotary files (Dentsply, Maillefer, Switzerland) according to the manufacturer’s instructions up to # F5. The file had a 0.5 diameter tip and variable taper along its length. For irrigation between successive instrumentation, 3 ml of 2.5% NaOCl (Clorox, Egypt) was delivered at a rate of 3 ml/min using a disposable plastic syringe with 30G side-ended needle (Sung Shim Medical Co., Bucheon, Gyeonggi, South Korea) 2 mm short of the total working length. At the end of the preparation, each canal received the final flush protocol with 5 ml of 2.5% NaOCl for 1 min, then 5 ml of 17% Ethylenediaminetetraacetic Acid (EDTA,Meta Biomed, Korea) for 1 min and 5 ml of saline (Novartis, Egypt) for 1 min to remove the smear layer [25]. All root canals were obturated with the cold lateral compaction technique using gutta-percha and AD-seal sealer (Meta Biomed, Korea). The quality of the obturation was confirmed radiographically. After obturation, 3 mm of gutta-percha was removed from the canal, and temporary filling material (Cavit,3 M ESPE, St. Paul, USA) was placed. All roots were kept in 100% humidity at 37 °C for 7 days to ensure complete setting of the sealer [25].

### Attachment fabrication

**Fig. 1:**
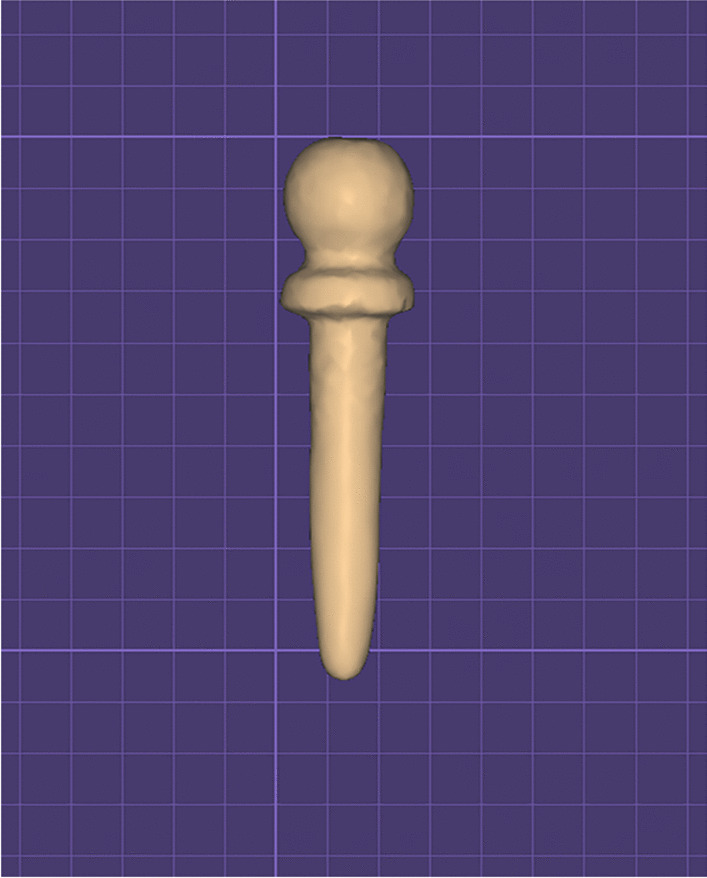
An image of the scanned ball attachment on the Exocad software to be milled into PEEK (polyetheretherketone) and PEKK (polyetherketoneketone)

For group TI, ready made titanium ball attachments (9 mm length OT Pivot flex, Rhein 83, Italy) were purchased. The titanium attachment was scanned (DOF swing scanner, DOFlabs, Seoul, South Korea) and a standard tessellation language (STL) file was generated using a CAD software (Exocad Dental CAD, Exocad Inc. Darmstadt, Germany) (Fig. 1). The file was used for milling similar ball attachments made of PEEK (Brecam Biohpp,Bredent, Germany) that were used in group PE. Similarly, the STL file was used for milling the PEKK ball attachments (Pekkton Ivory, Cendres^+^Metaux Medtech, swizerland) that were used in group PK [16].

### Root canal drilling and attachment cementation

After the 7-day setting period, preparation and cementation of the ball attachments were performed. A channel was created in each tooth for the ball attachments using the manufacturer’s drill (Mooser post reamer bur, Rhein 83, Italy). A single operator mounted the bur to a low speed handpiece to prepare the obturated canals to a length of 9 mm. The channels were rinsed with water and dried with paper points (Meta Biomed, Korea). EDTA was injected into the prepared space and left in place for 1 min to remove the smear layer. The canal space was then rinsed with saline and dried with paper points [25].

The post of each attachment was blasted with 50um aluminum oxide particles (BEGO sandblaster, BEGO Bremer GMBH, Germany) for 15 s at 0.25 MPa and 2–3 bars [14, 15, 20, 26, 27]. After sandblasting, the samples were dried with oil-free compressed air. The attachments in group TI were primed using Z-prime plus (Bisco Inc, United States of America) [26]. The attachments in the three groups were then inserted and cemented with self-adhesive resin cement (G-CEM, GC, Tokyo, Japan) following the manufacturer’s instructions. The attachments were inserted to the depth of the prepared channels with finger pressure then the excess cement was removed and light curing then followed. One trained operator cemented all the samples.

### Mechanical aging

#### Model creation for mechanical aging

For mechanical aging, an educational mandibular completely edentulous cast (Ramses edentulous cast; Ramses medical products, Cairo, Egypt) was used in this study. A waxed-up mandibular denture was made for this cast. The cast was then scanned (DOF swing scanner; DOFlabs, Seoul, South Korea) and a standard tessellation language (STL) file was generated. The cast with the overlying waxed up denture was then scanned and the STL file was generated. Both STL files were superimposed to determine the position of the mandibular canines’ sockets in the virtual model on the CAD software (Exocad Dental CAD; Exocad Gmbh, Darmstadt, Germany). Mandibular right and left acrylic canine teeth (Ramses medical products, Cairo, Egypt) were scanned and the STL file was generated. This STL file was then used for subtraction of the mandibular canines from their corresponding sockets in the previously scanned mandibular model. A space of 0.25 mm was left between the inner surface of the socket and the canine root surface simulating the periodontal membrane space. A 2 mm layer thickness was removed from the scanned model crest representing the mucosal layer that was added later [28]. The design of the virtual model was checked and the STL file was sent to the additive manufacturing device (ULTRA 3SP; EnvisionTEC Inc, Michigan, United States of America). The printed model was then duplicated into 15 similar acrylic resin models (HUGE Dental Material CO, Shandong, China) that were randomized and allocated into the three groups of the attachments used in the current study.

#### Mucosa and periodontal ligament simulation

The abutment teeth were ditched on their labial and lingual surfaces and then placed in their corresponding sockets in each model after injecting a light bodied rubber base material (AFFINIS™ light body, Coltene whaledent Inc., Altstätten, Switzerland) for periodontal ligament simulation [29]. The simulated periodontal ligament was then secured in place with a thin film of cyanoacrylate adhesive (Amir Alpha; Amir Alpha Co., Cairo, Egypt).

**Fig. 2:**
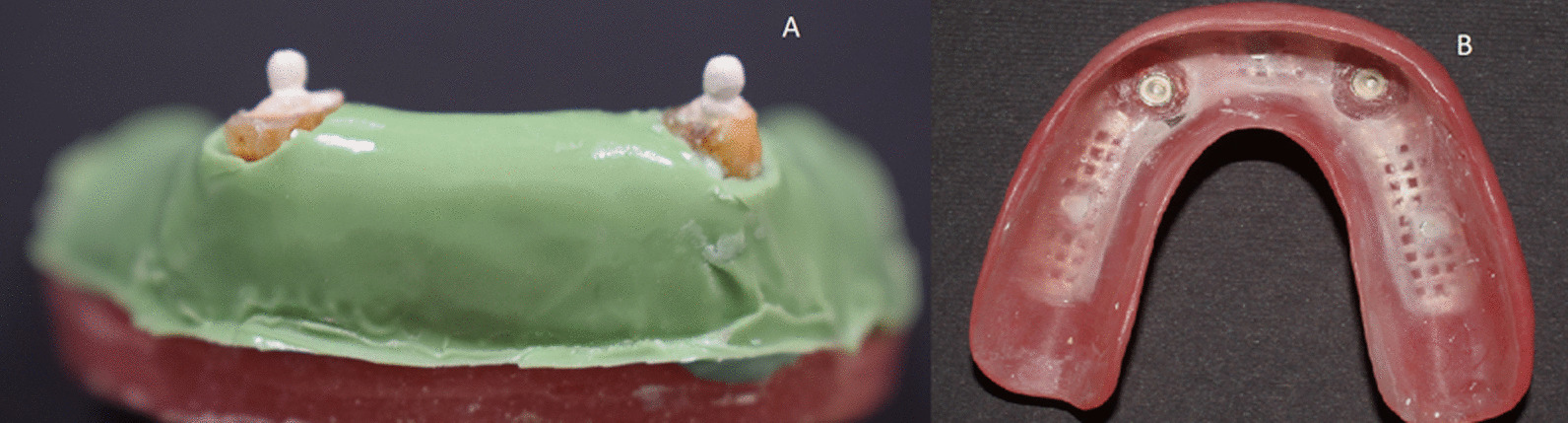
**a** A photo showing the model having the overdenture abutments with the ball attachments and simulated mucosa. **b** A photo of the overdenture intaglio surface showing the metal housing picked up in the reinforced base

For standardized mucosa simulation among the models, an acrylic template was used. A base plate wax of thickness 2 mm was added to the crest and slopes of the mandibular residual ridge in the printed model. Duplication of the model then followed to produce a stone model. The stone model was then placed in the vacuum press machine (Yates Motloid, United States of America) and an acrylic sheet (Bio-Art Equipamentos Odontologicos Ltda, Brasil) was pressed over it. The acrylic template was then trimmed and tried on the other models to check the fit. A light bodied rubber base impression material (AFFINIS™ light body, Coltene whaledent Inc., Altstätten, Switzerland) was dispensed in the acrylic template and seated over each model for mucosa simulation [28]. The simulated mucosa was then secured in place with a thin film of cyanoacrylate adhesive (Amir Alpha; Amir Alpha Co., Cairo, Egypt) (Fig. 2a).

#### Overdenture fabrication and determination of the geometric center

An overdenture was fabricated for the printed model and duplicated into 15 similar overdentures (Fig. 2b). For locating the geometric center on the printed model, two lines parallel to each other were determined; the first one passed through the apices of the retromolar pad and the second one passed through the incisal edge of the mandibular incisors. The midpoint on a third line that bisected the model and was perpendicular to the two previous lines represented the geometric center of the model [30]. A modeling wax sheet was then shaped in the form of a plate that was 10 mm in anterior–posterior dimension, 2 mm thickness and joining the occlusal surfaces of the teeth on both sides of the arch. The wax plate was then placed on the overdenture of the printed model so that its center was coincident with the geometric center of the arch. In the center of the wax plate, a recess was made to accommodate the tip of the load applicator in the chewing simulator machine. The wax plate was then invested (Bego bellavest^®^, Bego Gmbh, Bremen, Germany) and cast into a metal plate (Wironit, Bego Gmbh, Bremen,Germany). The metal plate was repositioned on the printed model in the previous predetermined position of the wax plate. An acrylic template was then made in the same way as that made for mucosa simulation in the current study. The acrylic template was then used to replicate the same position of the metal bar in the other models. The metal bars were positioned in place using autopolymerizing acrylic resin.

#### Aging and chewing simulation

**Fig. 3:**
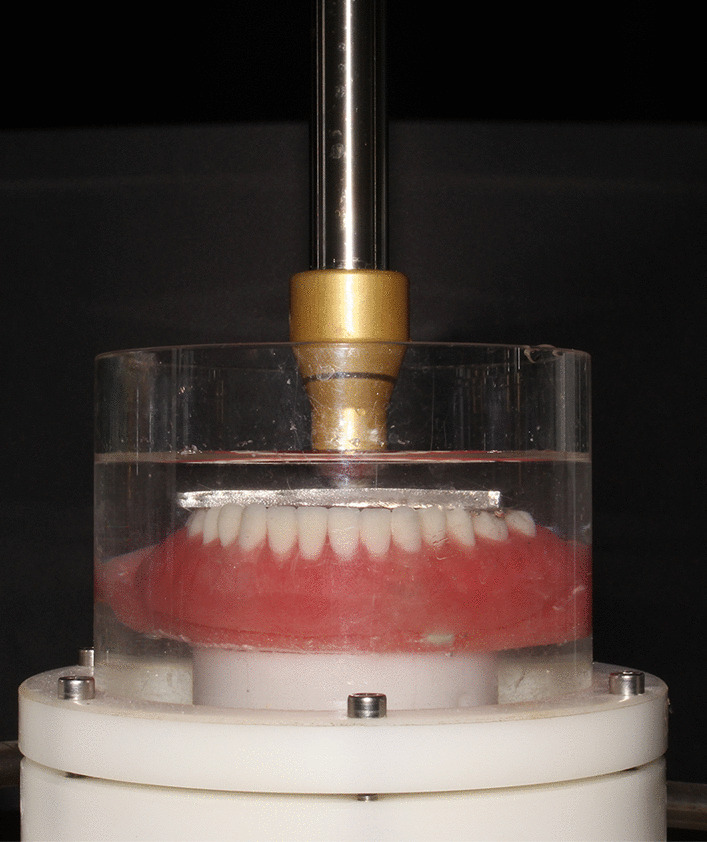
A photo of the overdenture mounted in the chewing simulator for mechanical aging

For aging, the chewing function in addition to overdenture insertion and removal were simulated in the current study. Each overdenture was inserted and removed 5000 times by one operator to simulate patient’s insertion and removal [31]. For chewing simulation, the mounting ring of the chewing simulator (CS-4.4; SD Mechatronic, Germany) was painted with Vaseline. Each model was then placed so that the load applicator was positioned in its predetermined position in the metal bar. The model was then secured in place with autopolymerizing acrylic resin (HUGE Dental Material CO, Shandong, China). The setting parameters of the chewing simulator were adjusted (60 mm/sec, 5 mm vertical path, 0.5 mm horizontal path, 1.6 Hz frequency, 68.6 N).The machine chambers were filled with artificial saliva prepared in the pharmaceutical industry lab in the author’s university according to the composition of (Glandosane^®^; Fresenius Kabi Ltd, Germany). Bi-axial cyclic loading of 1,200,000 cycles at room temperature were applied to each model simulating five years of service (Fig. 3). The teeth were then removed from the models for tensile bond strength testing.

### Samples preparation for testing

**Fig. 4:**
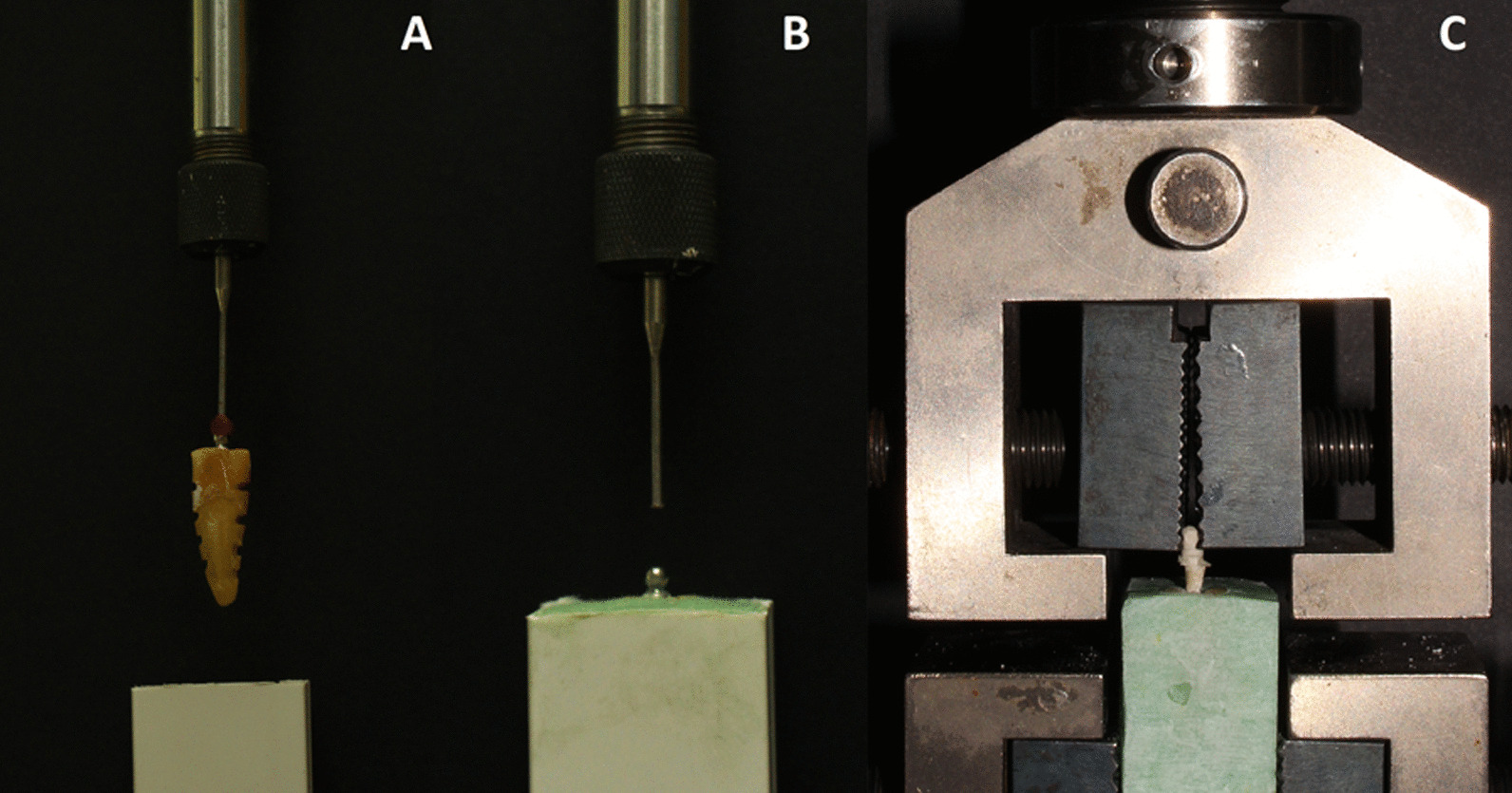
**a** A photo showing the overdenture abutment ditched along the labial and lingual surfaces of the root and attached to the analysing rod of the surveyor. **b** photo of the overdenture abutment mounted in the acrylic mould. **c** A photo showing the sample mounted in the universal testing machine during the pullout test

After a period of 2 weeks storage in distilled water at room temperature, the samples were placed in a standardized acrylic mold. To ensure parallel alignment of all the samples in their molds, the top part of the attachment was placed in line to the analyzing rod of a dental surveyor (Ney Surveyor, NeyTech, United States of America) by using sticky wax (Dentax ElKods,Cairo,Egypt) so that the long axis of the abutment was coincident with that of the analyzing rod (Fig. 4a). The vertical arm of the surveyor was then lowered to place the sample in the mold filled with the acrylic dough till the acrylic level just covered the shoulder of the sample coronal part (Fig. 4b). Placement of the samples in the molds was done by one trained operator.

The samples were mounted in the Universal Testing Machine (Lloyd LRX; Lloyd Instruments Ltd., Fareham, UK) for the pull out test. They were placed parallel to the loading direction and the machine grips grasped the attachment head. A constant loading rate of 0.5 mm/min was applied until failure was achieved. The load of failure was confirmed by a sharp drop in the load deflection curve recorded (Fig. 4c). Nexygen data-analysis software (Lloyd Instruments Ltd., Fareham, UK) was used for data display in Newtons [26, 27, 32, 33]. The recorded values were then divided by the bonding area to calculate the tensile bond strength value [16].

### Failure mode analysis

Failure mode analysis was determined by microscopic examination of the samples surface. Examination was done under 65X stereomicroscope (Olympus SZX16, OLYMPUS, Tokyo, Japan). Failures were classified into 1 of 3 possible categories: (1) adhesive failure either between the attachment and the resin cement or between the resin cement and root dentin (2) cohesive failure within the resin cement (3) mixed adhesive and cohesive failures [32, 34]. Failure mode was analyzed by one calibrated operator.

For blinding of the operators during tensile bond strength measurement and failure mode analysis, the samples were coded with secret codes by a colleague who decoded them after measurement.

### Statistical analysis

Data were checked using Kolmogrov-Smirnov test and showed normal distribution. One way ANOVA test followed by PostHoc Tukey test were used for intergroup comparisons. Paired T test was used for comparisons of the tensile bond strength pre and post aging in the same group. The significance level was set at P < 0.05 in all tests. Statistical analysis was performed using the statistical package for social sciences (version 21.0 SPSS Inc; IBM Corporation, Chicago, IL, USA).

## Results

**Fig. 5:**
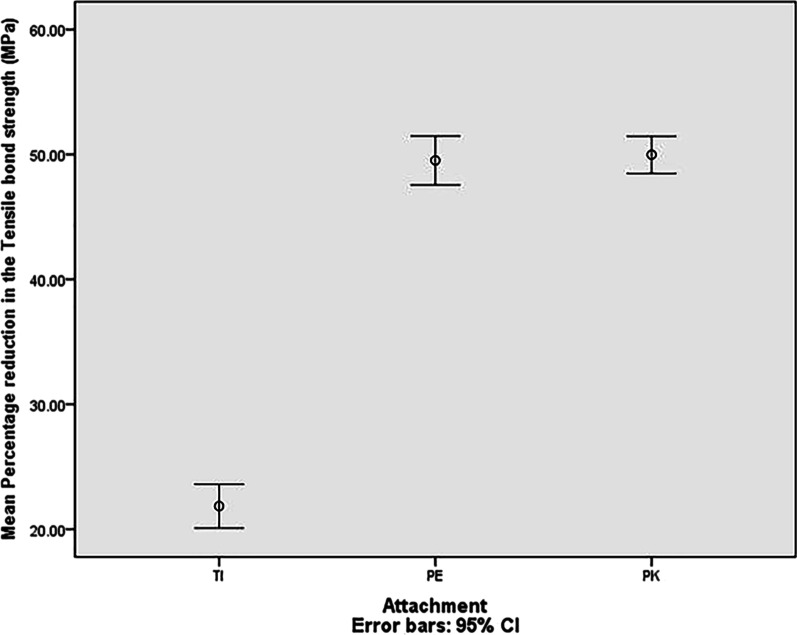
An image for the mean percentage reduction in the tensile bond strength of the three attachment groups (TI; titanium, PE; polyetheretherketone, PK; polyetherketoneketone) between T0 (pre aging) and T1 (post aging)

One way Anova test showed statistically significant difference between the three groups at the T0 timing. The PK group showed the highest tensile bond strength value needed to remove the ball attachment from the tooth root, while the TI group showed the lowest one. Post Hoc Tukey test showed statistically significant difference between the groups TI and PE (P < 0.00001) in addition to TI and PK (P < 0.00001). However, there was no statistically significant difference between the groups PE and PK (p = 0.13). Similar results were also attained for the three groups at the T1 timing as Post Hoc Tukey test showed statistically significant difference between the groups TI and PE (P < 0.00001), in addition to TI and PK (P < 0.00001), and statistically insignificant difference between the groups PE and PK (p = 0.84). The mean and standard deviation values are listed in Table 1. Paired T test showed statistically significant difference in the three groups on comparing the tensile bond strength at T0 and T1 intervals (Fig. 5). The mean and standard deviation values are listed in Table 2.

**Table 1:** Comparison of the mean and standard deviation values of the Tensile bond strength between the three groups pre and post aging

	TI		PE		PK		F value	P value
	X (MPa)	SD	X (MPa)	SD	X (MPa)	SD		
T0 (pre aging)	6.5	0.29^a^	18.96	0.37^b^	19.24	0.24^b^	5653.91	< .00001
T1 (post aging)	5.08	0.22^x^	9.57	0.32^y^	9.64	0.35^y^	720.21	< .00001

Different superscript lowercase letters (a, b) indicate significant difference between the three groups at T0. Different superscript lowercase letters (x, y) indicate significant difference between the three groups at T1

X, mean; SD, standard deviation; T0, pre aging measurement; T1, post aging measurement; MPa. Mega Pascal unit

**Table 2:** Comparison of the mean and standard deviation values of the tensile bond strength pre and post aging in each group

Attachment group					T value	P value
	T0 Pre aging		T1 Post aging			
	X (MPa)	SD	X (MPa)	SD		
Group TI	6.5	0.28	5.08	0.22	27.05	< .00001
Group PE	18.96	0.37	9.57	0.32	48.36	< .00001
Group PK	19.24	0.24	9.64	0.35	75.27	< .00001

X, mean; SD, standard deviation; T0, pre aging measurement; T1, post aging measurement; MPa, Mega Pascal unit

The ball attachments showed a mixed mode of failure in the three groups with a predominant adhesive failure at the cement interface. In the group TI, the adhesive failure was mainly between the attachment surface and the cement. However, in the groups PE and PK, the adhesive failure was between the cement and the dentin surface.

## Discussion

The remaining dentition in the elderly people may offer poor support for the conventional removable partial dentures. Suitable abutments can be selected and prepared to receive a tooth supported overdenture that helps to improve retention, reduce the rate of bone resorption and maintain proprioception. Canine teeth are usually selected as overdenture abutments because they have strong roots, lie in the corner of the mouth, reduce the rate of bone resorption in the anterior segment and their root canal treatment is easily manged [1, 2]. Ball stud attachments are among the commonly used ones in tooth supported overdentures [1]. However, most of the commercially available ones are made of Titanium and debonding of such attachments are reported in the literature [8, 9]. So, the purpose of the current study was to evaluate the tensile bond strength of such attachments made of Titanium, PEEK and PEKK when bonded to root dentin under thermal and mechanical simulated aging.

The null hypothesis was rejected in this study as there was a statistically significant difference between the three groups before and after aging. The PEEK and PEKK groups showed higher tensile bond strength values than the titanium group while there was no difference between the two members of the PAEK family as revealed by Post Hoc test. Such a result comes in line with another studies in which pretreated PEEK surface coupled with self adhesive resin cement showed higher tensile bond strength compared to titanium [13, 16]. Furthermore, PEKK posts also showed higher tensile bond strength values compared to the conventional posts [27]. However, the results of the current study do not match the results published in another study whereas PEEK showed higher tensile bond strength values than PEKK. This may be related to the type of PEEK used in that study being a fibre reinforced one. The presence of fibres in the fibre reinforced PEEK resulted in more surface roughness compared to PEKK; a condition that improved micromechanical bonding with the adhesive [15].

The attachment posts in the current study received an air borne abrasion with alumina particles to improve the tensile bond strength. PAEK surfaces that received air borne abrasion showed higher bond strength values than those that did not receive air borne abrasion as stated in the literature [13, 14, 20, 24, 27, 35]. A study highlighting the effect of air borne abrasion showed that there was no synergistic effect of Piranha acid pretreatment in the presence of air borne abrasion for PEEK surfaces [11, 20]. Moreover, PEEK surfaces that solely received piranha acid treatment showed no adhesion with resin cements [11]. Further study calling the attention to the importance of air borne abrasion revealed that the different priming systems did not have a noticeable effect on the tensile bond strength of air abraded PEEK crowns when cemented to dentin abutments. However, such systems led to a stronger tensile bond strength when the PEEK crowns received Piranha acid pretreatment [20]. The same study also showed that either etching PEEK surface with strong acids as 98% sulphuric acid or air borne abrasion had the same effect on the tensile bond strength values which were significantly higher than the values attained by Piranha acid in the same study [20]. Similar results were also found for PEKK substrates pretreated with 98% sulphuric acid and air borne abrasion [24, 27]. Moreover, chair side treatment of the attachments with 98% sulphuric acid is not practical and dangerous. Moreover, coupling air borne abrasion with the presence of monomers in adhesive resins helped to achieve the highest bond strength value even after aging and thermocycling [11, 12, 19]. That’s why air borne abrasion was selected for pretreatment of the attachments before cementation in the current study.

Air borne abrasion improves adhesion by increasing the surface area available for physical bonding and allowing micromechanical retention [13, 14, 21, 24, 27, 34–36]. Microroughness of the air borne abraded surface was another reason that improved the contact area between the functional groups of the adhesive and the substrate surfaces [14, 16, 24, 26, 27, 37].

A further reason for the higher tensile bond strength of PEEK and PEKK in the current study is the used cement that is based on phosphoric esters and functional monomers (4-MET). The presence of functional monomers may have helped to increase the bond strength of pretreated PEEK and PEKK as mentioned in the literature [11, 12, 19]. Further study showed that the application of adhesives containing multifunctional methacrylates for cementation of air abraded PEEK resulted in higher bond strength values compared to other types of adhesives. Such high tensile bond strength values were attributed to the strong chemical bond between the pretreated PEEK surface and the multifunctional methacrylate molecule [13, 22]. The phosphoric ester as a part of the adhesive system may also have played a role to improve the bond strength as it showed the highest bond strength when coupled with air borne particle abrasion for cementation of PEEK crowns to teeth compared to other adhesive systems used for cementation [20]. The nature of the adhesive resin cement also affects the spreading coefficient of the adhesive in addition to the interfacial surface tension and the work of adhesion between the adhesive and the substrate [22, 23]. The cement used in the current study was hydrophilic, had low viscosity and an acidic P^H^ as stated by the manufacturer. Such properties may have improved flow and penetration into the dentinal tubules. The low viscosity of the adhesive system was also a reason mentioned to improve the tensile bond strength as it facilitated infiltration of the adhesive into the air abraded surfaces and dentinal tubules allowing strong bonding [22, 23]. The deep infiltration of the cement in addition to the improved contact with the roughened surface allowed deeper and wider area of chemical bonding between the functional monomer of the adhesive and the substrate [22]. The low P^H^ value of the cement used in this study may have allowed dentin demineralization, better monomer diffusion leading to more favourable condition for copolymerization and formation of strong chemical bonds with the root dentin [38]. The acidity of the cement may also have altered the chemical characteristics of PEEK and PEKK surfaces and improved their surface polarity by breaking the benzene ring and exposing more functional groups for chemical bonding with the functional monomers of the adhesive [13, 14, 20, 38].

However, the acidity of the cement did not have a similar effect on the Titanium surfaces that require an alkaline treatment rather than an acidic one for effective chemical bonding [26, 37]. Alkaline treatment following air borne abrasion of titanium substrates results in a chemical bonding between the hydroxyl group of the alkalinized surface and the acidic groups of the adhesive resin [26, 37]. So, the lower tensile bond strength of the titanium attachments could be related to the nature of bonding that occurred in the current study that was a micromechanical physical one and the absence of the strong chemical bonds that occurred in the PEEK and PEKK groups in addition to the micromechanical physical one. Moreover, Yang et al. stated that the metal and ceramic primers as that used in the current study compared to the metal primers did not have a noticeable effect on the tensile bond strength between the adhesive resin and the Titanium substrate [26]. They rationalized such a finding in the light of the concentration of the functional monomer in the primer that was below 10% of the molecular weight of the primer. However, for the primer to achieve an effective bonding between adhesive resin and Titanium surfaces, the functional monomer must compose more than 10% of the primer’s molecular weight [26, 39].

Paired T test showed statistically significant reduction in the tensile bond strength with aging in the three groups. Fuhrmann et al. and Cao et al reported similar results for the PAEK family and Titanium substrates respectively [15, 26]. The decrease in the tensile bond strength by aging could be attributed to the stresses developed at the bonding interface and crack propagation [15, 26]. Furthermore, the fluid added during simulated chewing may have caused hydrolysis of the bonds with consequent weakening of the tensile bond strength between the adhesive and the substrate.

Examination of the samples after failure revealed that the titanium attachments had a mixed mode of failure with a predominant failure of adhesion between the attachment surface and the cement. On the other hand, PEEK and PEKK attachments showed a mixed mode of failure with a predominant failure of adhesion between the cement surface and the root dentin. Such results are consistent with the results of the tensile bond strength in the current study. The high tensile bond strength values in the groups PE and PK reflected more frequency of adhesive failure between the cement surface and the root dentin rather than failure between the attachment surface and the cement that occurred in group TI; the one with lower tensile bond strength values. Similar findings were reported in further studies [15, 22]. Moreover, pretreatment of PAEK with air abrasion or sulphuric acid resulted in failure between the cement surface and dentin rather than failure between the PAEK surface and the cement that occurred in non pretreated PAEK even in the presence of adhesive systems as reported in the literature. Such finding was attributed to the positive effect of PAEK pretreatment before cementation on the tensile bond strength as mentioned earlier [14, 15, 20, 22]. On the other hand, the ball attachments in the group TI showed low tensile bond strength values with consequent failure between the attachment surface and the cement. Such a result came in line with Benli et al. who reported failure of adhesion between the post surface and the cement for titanium posts rather than failure between the cement surface and the dentin for the PEEK posts [16].

Regarding the clinical setup of the current study results, the PAEK ball attachments may have a lesser incidence rate of debonding compared to the Titanium ones. Moreover, the PAEK attachments owing to their shock absorption and their dentin matching elastic modulus, could be speculated to have a lesser rate of vertical root fractures compared to the titanium ones; a condition reported for the dental posts made of the same materials. Accordingly the lesser the complications prospected for the PAEK ball attachments, the reduced the patient apprehension.

Most of the studies in the literature compared the tensile bond strength and the failure mode analysis of the PAEK family members and titanium in the different dental applications as dental posts and crowns. However, none of them referred to such an issue in the overdenture ball attachments; a strength point in the current study. Moreover, mechanical simulated chewing aging was mimicked in the current study, but not in the previous studies. Furthermore, the authors managed to control variables as one investigator made the root canal treatment and cemented the attachments for all the samples. Acrylic templates were also used to standardize the mucosa thickness and the chewing load application. Light body rubber base material was used for mucosa and periodontal ligament simulation for more natural simulation and realistic results as stated by Brosh et al. [29]. On the other hand, a number of limitations are available in the current study as no alkaline treatment was made to the Titanium attachments and a universal primer instead of a metal primer was used during their cementation; a condition that may have improved bonding. Moreover, the PEEK and PEKK attachments in the current study were of similar size to that of Titanium yet they are of lower strength values. Clasps in the PEEK and PEKK removable partial dentures were also made thicker than the conventional metallic ones for additional strength [17, 18]. So, further studies investigating the appropriate size of PEEK and PEKK attachments are recommended to validate their application. Preclinical investigations evaluating the fracture strength of PAEK attachments in comparison to titanium ones should be made as well. Randomized clinical trials comparing the clinical performance of PAEK and Titanium stud attachments should be designed too.

## Conclusion

Inspite of the current study limitations, PEEK and PEKK overdenture ball attachments could be concluded to have a higher tensile bond strength compared to titanium ones when bonded to root dentin. Tensile bond strength of such attachments may decrease with aging as well.

## Data Availability

The dataset supporting the conclusions of the current article are provided as a supplementary material in an additional file.

## Abbreviations

PAEK: Polyaryletherketone
PEEK: Polyetheretherketone
PEKK: Polyetherketoneketone
STL: Standard tessellation language
MPa: Mega Pascal
